# A Population of Projection Neurons that Inhibits the Lateral Horn but Excites the Antennal Lobe through Chemical Synapses in *Drosophila*

**DOI:** 10.3389/fncir.2017.00030

**Published:** 2017-05-03

**Authors:** Kazumichi Shimizu, Mark Stopfer

**Affiliations:** National Institute of Child Health and Human Development, National Institutes of Health Bethesda, MD, USA

**Keywords:** olfaction, *Drosophila*, antennal lobe, electrophysiology, electrical synapses, chemical synapses, GABA, lateral excitation

## Abstract

In the insect olfactory system, odor information is transferred from the antennal lobe (AL) to higher brain areas by projection neurons (PNs) in multiple AL tracts (ALTs). In several species, one of the ALTs, the mediolateral ALT (mlALT), contains some GABAergic PNs; in the *Drosophila* brain, the great majority of ventral PNs (vPNs) are GABAergic and project through this tract to the lateral horn (LH). Most excitatory PNs (ePNs), project through the medial ALT (mALT) to the mushroom body (MB) and the LH. Recent studies have shown that GABAergic vPNs play inhibitory roles at their axon terminals in the LH. However, little is known about the properties and functions of vPNs at their dendritic branches in the AL. Here, we used optogenetic and patch clamp techniques to investigate the functional roles of vPNs in the AL. Surprisingly, our results show that specific activation of vPNs reliably elicits strong excitatory postsynaptic potentials (EPSPs) in ePNs. Moreover, the connections between vPNs and ePNs are mediated by direct chemical synapses. Neither pulses of GABA, nor pharmagological, or genetic blockade of GABAergic transmission gave results consistent with the involvement of GABA in vPN-ePN excitatory transmission. These unexpected results suggest new roles for the vPN population in olfactory information processing.

## Introduction

**Figure 1:**
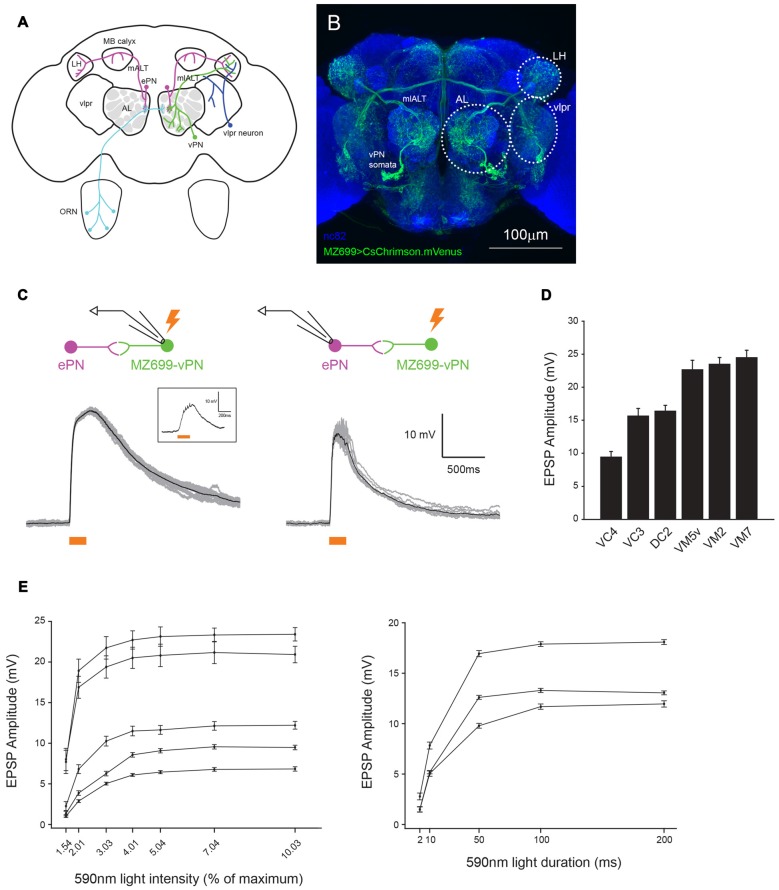
**MZ699+ neurons send glomerulus-specific excitation to excitatory projection neurons (ePNs). (A)** Schematic representation of the *Drosophila* olfactory system. Two large neural populations in different brain areas, ventral PNs (vPNs; green) and ventrolateral protocerebrum (vlpr) neurons (blue) express *MZ699-Gal4*. **(B)** A confocal stack image of CsChrimson.mVenus expression driven by *MZ699-Gal4*. The neuropils were visualized by staining with nc82 antibody (blue). **(C)** Optogenetic activation of *MZ699-Gal4* neurons with ~64 μW/mm^2^ 200 ms whole-field 590 nm light elicited large depolarizations well above the spiking threshold in a MZ699 vPN (left); the average (black) of 10 trials (gray) from an example MZ699-vPN. The spikes the recorded neuron produced were small and difficult to see in the raw traces. A raw voltage trace of the same neuron upon stimulation with ~1.3 μW/mm^2^ light for 200 ms is shown in the inset. A stronger light stimulus (~64 μW/mm^2^ 200 ms whole-field 590 nm light) delivered to the brain elicited large and reliable excitatory postsynaptic potentials (EPSPs) in the recorded ePNs (right); the average (black) of 10 trials (gray) from an example ePN in VM5v glomerulus is shown. **(D)** The magnitude of excitation in ePNs upon *MZ699-Gal4* > CsChrimson neurons was glomerulus-specific (mean ± SEM, *n* = 4 for VC4, *n* = 5 for VC3, *n* = 3 for DC2, *n* = 4 for VM5v, *n* = 4 for VM2, *n* = 2 for VM7). Recorded ePNs were filled with dye, and their glomeruli were identified by comparing their positions with a standard atlas (Yu et al., 2010). **(E)** The magnitude of excitation in *MZ699-Gal4* > CsChrimson ePNs varies with the intensity and duration of the light stimulus. The glomerular identities of the recorded ePNs are as follows: from the ePN with largest amplitude at 10.03%, VM5v, VM3, DC2, VA1d and DM6, and from the ePN with largest amplitude at 200 ms, VA1d, VC4 and VC3.

The insect antennal lobe (AL) is a useful model system to study neural computations. *Drosophila* has been a particularly beneficial model system because it offers numerous genetic tools for labeling and manipulating the activity of neurons. In the *Drosophila* olfactory system, olfactory receptor neurons (ORNs) on peripheral appendages detect odorants and transfer this information to the AL. There, axon terminals of ORNs synapse onto projection neurons (PNs) including the ePNs (Liang and Luo, 2010; Rytz et al., 2013), which then transmit odor information to the mushroom body (MB) and the lateral horn (LH) through the medial antennal lobe tract (mALT, Figure 1A). Each ORN expresses a single type of olfactory receptor (Vosshall et al., 2000) together with a non-odorant binding coreceptor (Orco, also known as Or83b, Larsson et al., 2004; Neuhaus et al., 2005). ORNs expressing the same receptor type project to the same glomerulus in the AL (Couto et al., 2005). Each ePN also projects its dendrites to a single glomerulus (Wong et al., 2002). Thus the identity of each ORN and PN can be defined by the glomerulus they target. ePNs have been studied extensively because they comprise the largest excitatory neural population in the *Drosophila* AL that sends information to other brain areas (Wilson, 2013).

Here we focus on a less studied population of PNs, the ~50 vPNs, which can send uniglomerular, multiglomerular or pan-AL dendritic projections (Figure 1A, Lai et al., 2008). *MZ699-Gal4* is expressed in about 90% of all vPNs, which can be uniglomerular or multiglomerular (Figure 1B, MZ699-vPNs hereafter, Lai et al., 2008). Of these MZ699-vPNs, about 80% have been shown to be GABAergic by *in situ* hybridization against *Gad1* (Okada et al., 2009); it is not known which neurotransmitter is expressed by the remaining 20% of MZ699-vPNs. MZ699-vPNs project through the mediolateral antennal lobe tract (mlALT) only to the LH and are presumed to inhibit follower neurons. Indeed, the axon terminals of these MZ699-vPNs have been reported to inhibit their postsynaptic LH neurons (LHNs) through GABAergic synaptic transmission (Liang et al., 2013). On the other hand, very little is known about the functional roles dendritic arbors of vPNs play in the AL except that they have been reported to weakly excite ePNs through electrical synapses (Wang et al., 2014).

To better understand this excitatory transmission, we combined whole-cell patch clamp recordings with pharmacological and optogenetic manipulations to characterize connections from MZ699-vPNs onto ePNs. Surprisingly, our results show that MZ699-vPNs strongly, reliably, and monosynaptically excite the ePNs through chemical synapses. Our results suggest novel information processing roles for the second largest group of olfactory PNs in the AL.

## Materials and Methods

### Fly Strains

**Figure 2:**
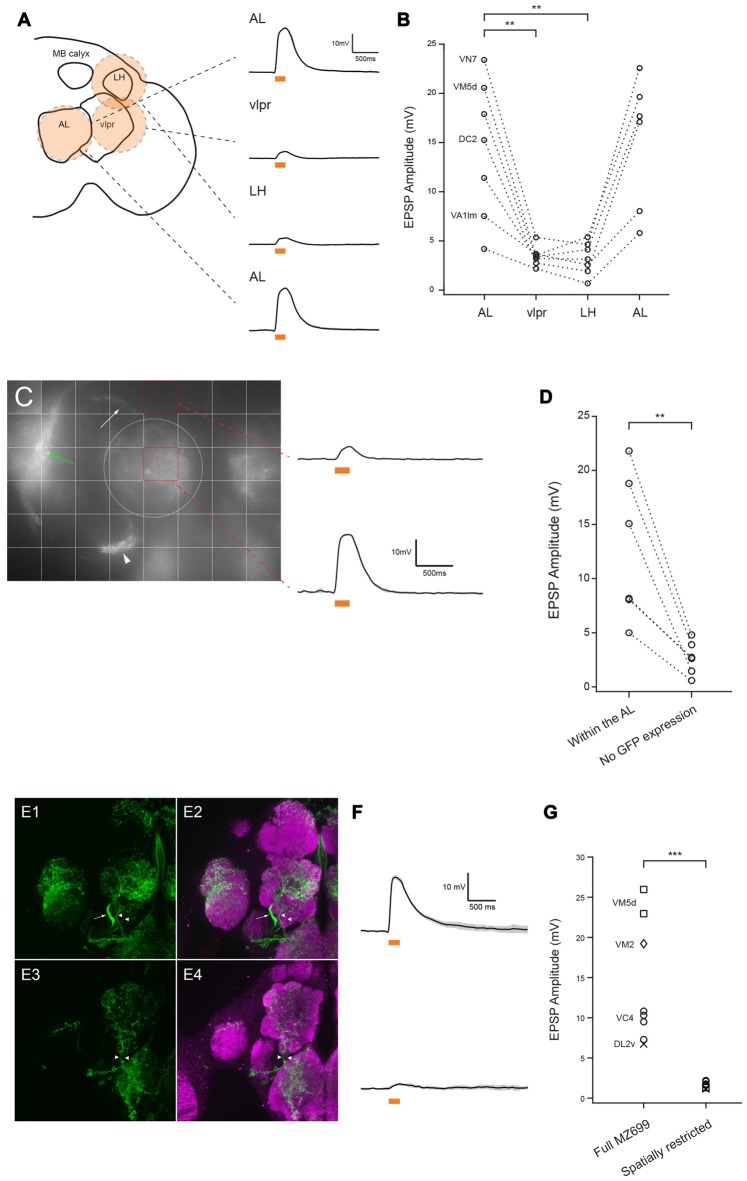
**MZ699-vPNs are the source of excitation to the ePNs. (A)** Schematic illustration shows how a circular flash of 590 nm light was directed sequentially to the antennal lobe (AL), lateral horn (LH), vlpr and then back to the AL. Example traces from a VM7 ePN are shown on the right (mean ± SD). **(B)** All recorded ePNs showed the greatest depolarization when 590 nm spot was directed to the AL (*n* = 7, paired *t*-test, **p < 0.01). The identities of four ePNs filled with biocytin hydrazide are shown on the left. **(C)** Grid stimulation of areas devoid of CsChrimson expression (e.g., upper red square) induced much smaller depolarization in an ePN (mean ± SD) than stimulation of areas including the AL (e.g., lower red square). The bundle of MZ699-vPNs projecting to the LH, the MZ699-vPNs’ somata, and the vlpr neurons are indicated with a white arrow, a white arrowhead and a green arrow, respectively. The AL is encircled with a white dotted line. **(D)** In all six ePNs, stimulation of an area without CsChrimson expression induced smaller depolarization than stimulation of areas including the AL (**p < 0.01, paired *t*-test). The glomerular identities of recorded ePNs are as follows: from the ePN with the largest magnitude with stimulation of AL, VM7, VM2, VM2, DC2, DC2 and VA1d. **(E1,E2)** *MZ699-Gal4* drove expression of CsChrimson (green) in vPNs (an axon bundle indicated with an arrow) and two fibers innervating the AL (arrowheads). **(E3,E4)** A genetic intersection strategy (crossing *MZ699-Gal4* and *Gad1-LexA::QFAD*) eliminated expression of CsChrimson selectively in the vPNs while expressing CsChrimson specifically in the two fibers (arrowheads). **(F)** Example voltage traces from two ePNs in the same type of glomerulus (VM5d). Top: full *MZ699-Gal4*; Bottom: spatially restricted genotype (mean ± SD). **(G)** Compared to results from the full *MZ699-Gal4* group, depolarization magnitude was much smaller when expression of CsChrimson in vPNs was eliminated from *MZ699-Gal4* expression pattern (*n* = 8 for control group, *n* = 7,1.68 ± 0.14 mV for experimental group, ***p < 0.001, two-way analysis of variance (ANOVA)). This result shows that, of the neuron populations tested, only MZ699-vPNs provide excitation to the ePNs.

**Figure 3:**
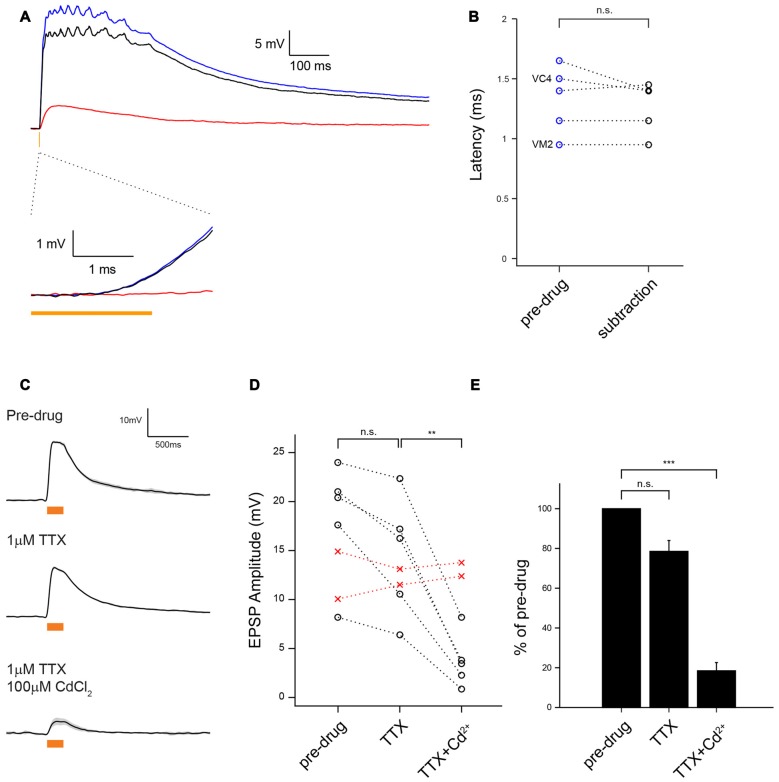
**MZ699-vPNs monosynaptically excite ePNs through chemical synapses. (A)** Average voltage traces of ePN depolarization on optogenetic activation of *MZ699-Gal4*^+^ neurons with 2 ms 590 nm pulse before (blue) and after perfusion with Cd^2+^ to block chemical transmission (red); the subtraction trace (black) reveals the chemical synaptic transmission component of the response. **(B)** The EPSPs latency from the light onset was very brief, consistent with a monosynaptic chemical connection (*n* = 5,1.33 ± 0.13 ms before Cd^2+^, 1.27 ± 0.10 ms after Cd^2+^, *p* = 0.32). The identities of two ePNs filled with biocytin hydrazide are shown on the left.** (C)** Example traces showing depolarization of an ePN induced by optogenetic activation of *MZ699-Gal4*^+^ neurons before drug application (top), after infusion of 1 μM tetrodotoxin (TTX; middle), and after subsequent infusion of 1 μM TTX and 100 μM Cd^2+^ (bottom). **(D)** Depolarization magnitude was slightly reduced with 1 μM TTX (*n* = 5, *p* = 0.027, paired *t*-test) but greatly reduced with subsequent infusion of 1 μM TTX and 100 μM Cd^2+^ (*n* = 5, **p < 0.01, paired *t*-test). Replacement of external saline with a control drug-free saline did not cause any change in EPSP amplitude (red crosses). The glomerular identities of the recorded ePNs are as follows: from the ePN with largest amplitude in pre-drug condition, VM3, VC3, D, DC2 and VA1d for the drug application group (black circle), and VC3 and VA2 for the drug-free saline group (red circles). **(E)** A bar graph shows the EPSP amplitude normalized by pre-drug responses (*n* = 5, TTX: 78.54 ± 5.45%, *p* = 0.017, TTX + Cd^2+^: 18.48 ± 4.13%, ***p < 0.001, paired *t*-test).

**Figure 4:**
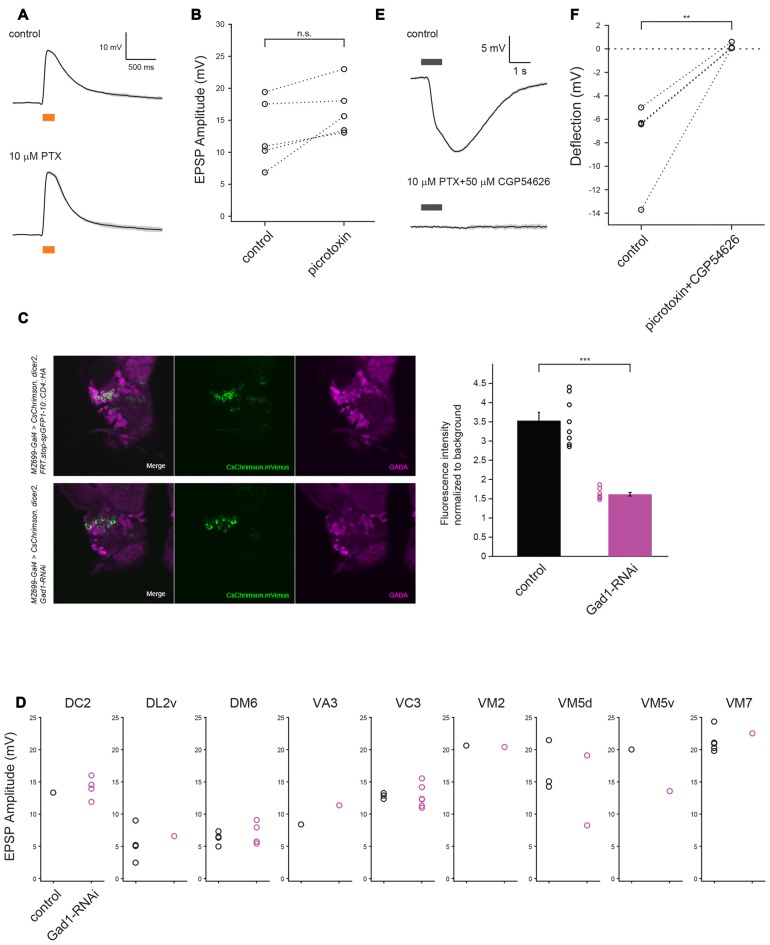
**GABA may not contribute to vPN-ePN excitatory synaptic connections. (A)** Example traces show EPSPs in ePNs triggered by optogenetic activation of vPNs before (top) and after (bottom) application of GABA_A_ blocker 10 μM PTX (mean ± SD), both in the presence of 1 μM TTX to block indirect activation of the neurons. **(B)** Perfusion with 10 μM PTX did not change the depolarization magnitude in any of five experiments (*n* = 5, *p* = 0.059, paired *t*-test). The glomerular identities of recorded ePNs are as follows: from the ePN with largest amplitude in control condition, VM5v, VM3, VA1d, VC4 and VC4. **(C)** RNAi mediated knock-down of *Gad1* with *MZ699-Gal4* resulted in decreased expression of GABA in MZ699-vPNs. Decrease in GABA staining signal in the vPN somata was more than two-fold (*n* = 8 hemispheres for control and *n* = 8 hemispheres for RNAi animals, ***p < 0.001, *t*-test). **(D)** Diminishing GABA expression by *Gad1* knock-down driven by *MZ699-Gal4* did not change EPSP magnitude of ePNs in nine glomeruli upon optogenetic activation of *MZ699-Gal4*^+^ neurons (*n* = 25 for control: black circles, *n* = 21 for RNAi: red circles, *p* = 0.59, two-way ANOVA). **(E)** Example traces show that puffs of 1 mM GABA delivered to the AL before (top) and after (bottom) bath application of 10 μM PTX and 50 μM CGP54626 (mean ± SD), both in the presence of 1 μM TTX, did not elicit depolarizing responses in ePNs; similar results obtained from five experiments are shown in **(F)** (The responses of three ePNs in the control group and four ePNs in experimental group were so similar that the plots for these cells are superimposed **p < 0.01, paired *t*-test).

**Figure 5:**
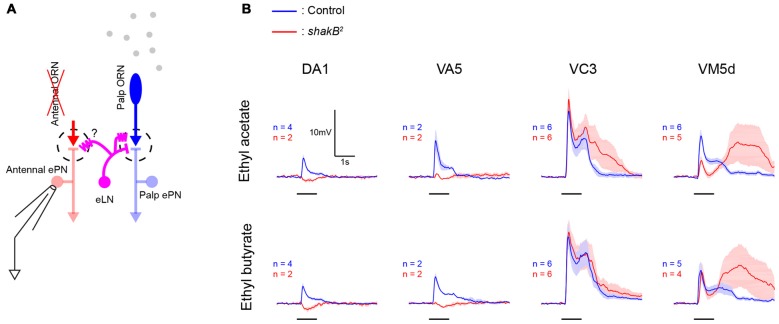
***shakB^2^* mutation does not abolish lateral excitatory inputs in all glomeruli. (A)** A schematic of the antenna-less preparation. Both antennae were removed immediately before experiments, maxillary palps were stimulated with odors, and patch recordings were performed from antennal ePNs lacking direct afferent inputs. **(B)** In antenna-less wild-type flies, odors evoked lateral excitation in ePNs in these four glomeruli (blue, mean ± SEM). While in DA1 and VA5 glomeruli of antenna-less *shakB^2^* mutant flies, these odor-evoked lateral excitatory inputs were abolished and inhibition dominated, ePNs in VC3 and VM5d of *shakB*^2^ mutant flies showed large lateral excitatory inputs (red, mean ± SEM).

**Figure 6:**
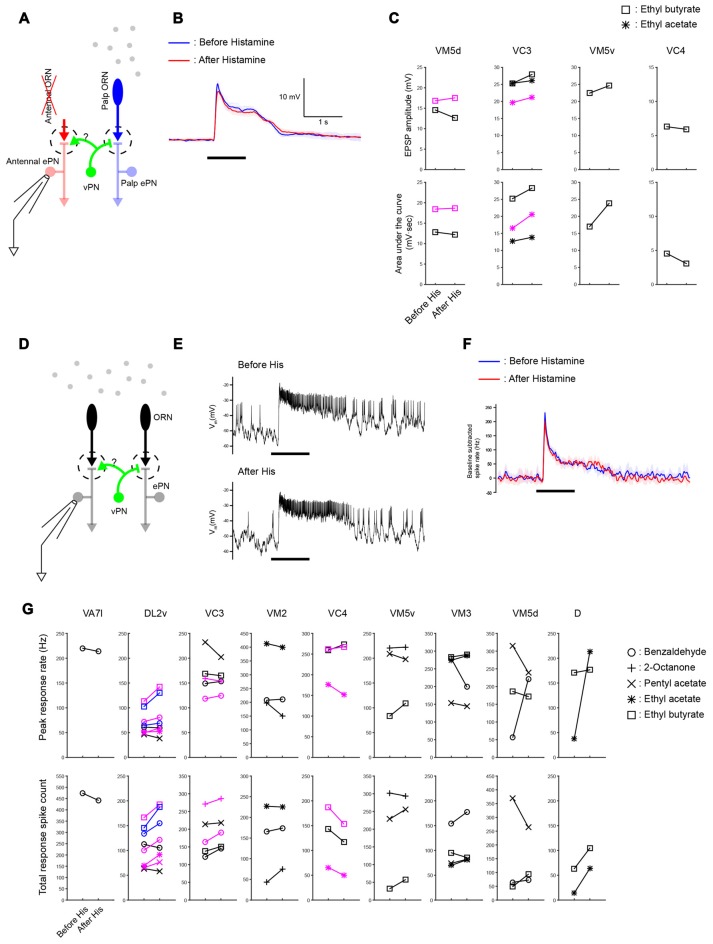
**Widespread lateral excitation is not affected by suppressing vPNs. (A)** A schematic of the antenna-less preparation. **(B)** Across-trial average of odor-elicited EPSPs from an example VM5d ePN (black plots in VM5d panel **(C)**) before and after suppressing vPNs expressing shunting His-Cl channels by adding histamine to the bath (mean ± SD) suggests that the vPN-ePN synapse does not contribute to lateral excitation. Odor stimulus in this example was a 1 s puff of 0.3% ethyl butyrate. **(C)** Results from similar experiments (six ePNs, seven odor-ePN pairs, *p* = 0.23 for the peak amplitude, *p* = 0.14 for the area under the curve, two-way ANOVA). Different ePNs in each glomerulus are indicated with different colors (black or magenta).** (D)** A schematic of the intact preparation. Both antennae and maxillary palps were stimulated with odors while ePNs were monitored with patch recordings. **(E)** Example voltage traces show odor-elicited responses in a VC3 ePN (black plots in VC3 panel **(G)**) before and after vPNs were suppressed by His-Cl channels. Odor stimulus was 1 s of 0.3% pentyl acetate. **(F)** Across-trial average of PSTHs from the same VC3 ePN shown in (**E**; mean ± SD). **(G)** Odor-elicited spiking responses in the palp ePNs (VA7l) and antennal ePNs (all others) were not reduced when vPNs were suppressed by His-Cl channels (13 ePNs, 32 odor-ePN pairs, *p* = 0.53 for the peak response rate, *p* = 0.13 for the total response spike count, two-way ANOVA, see “Materials and Methods” Section for response calculations). Different ePNs in each glomerulus are indicated with different colors (black, magenta or blue).

Fly stocks were maintained on standard *Drosophila* medium at ~25°C under a 12 h light/dark cycle. The fly strains we used are as follows: *MZ699-Gal4* (gift from T. Lee); *20xUAS-IVS-CsChrimson.mVenus attP2* (Bloomington #55136); *Gad1^MI09277^-LexA::QFAD* (gift from B.H. White); *LexO-FLP* (gift from K. Basler), *tubP(FRT.stop)Gal80* (Bloomington #38878), *UAS-Gad1-RNAi* (VDRC #32344), *GH146-QF, QUAS-mCD8::GFP* (Bloomington #30038), UAS-Dcr2 (Bloomington, #24646), *20xUAS-IVS-FRT.stop-spGFP1–10::CD4::HA*, *shakB*^2^ (gift from C.J.H. Elliott), *UAS-2HA.ort* (gift from C.H. Lee). The genotypes of experiments in this study and brief descriptions of each genotype are as follows:

- Figures 1, 2A,B,C,D,E1,E2,F (control), **G** (control), Figures 3, 4A,B,E,F: *yw/w;MZ699-Gal4/20xUAS-IVS-CsChrimson.mVenus attP2* (Red shifted channel rhodopsin (CsChrimson) was expressed in *MZ699-Gal4* positive cells).
- Figures 2E3,E4,F (experimental), **G** (experimental): *yw/w;LexO-FLP/tubP(FRT.stop)Gal80;MZ699-Gal4, 20xUAS-IVS-CsChrimson.mVenus attP2/Gad1^MI09277^-LexA::QFAD* (*MZ699-Gal4* activity was suppressed by Gal80 only in *Gad1* expressing cells, and expression of CsChrimson was lost in MZ699-vPNs as a result).
- Figures 4C (RNAi), **D** (RNAi): *w, UAS-dicer2/w;;MZ699-Gal4, 20xUAS-IVS-CsChrimson.mVenus attP2/UAS-Gad1-RNAi* (*MZ699-Gal4* drove the expression of CsChrimson, double strand RNA against *Gad1* and Dicer2. Dicer2 was expressed to enhance the efficiency of RNAi knock down).
- Figures 4C (control), **D** (control): *w, UAS-dicer2/w;;MZ699-Gal4, 20xUAS-IVS-CsChrimson.mVenus attP2/20xUAS-IVS-FRT.stop-spGFP1–10::CD4::HA* (As a control for sequestration of Gal4 molecules, *UAS-Gad1-RNAi* was substituted with *20xUAS-IVS-FRT.stop-spGFP1–10::CD4::HA*).
- Figure 5 (Control): *yw/+;;GH146-QF, QUAS-mCD8::GFP/+* (*GH146-QF* expressed mCD8::GFP marker in about two-thirds of ePNs).
- Figure 5 (shakB): *shakB*^2^ *or shakB^2^;;GH146-QF, QUAS-mCD8::GFP/+* (homozygotes of *shakB*^2^ mutation, which abolishes electrical synapses between excitatory local neurons (eLNs) and ePNs).
- Figure 6: *yw;UAS-2HA.ort/+;MZ699-Gal4/GH146-QF, QUAS-mCD8::GFP* (*MZ699-Gal4* drove expression of histamine gated chloride channels (ort). ePNs were labeled with mCD8::GFP driven by *GH146-QF*).

### Whole-Cell Patch Clamp Recordings

Patch-clamp electrodes (5–10 MΩ) were pulled from borosilicate glass pipettes (Sutter Instrument) and filled with the following internal solution: (in mM); 125 potassium aspartate, 10 HEPES, 4 MgATP, 0.5 Na3GTP, 1 EGTA, 1 KCl, pH adjusted to ~7.3 with KOH. To morphologically characterize the recorded cells, in most recordings ~6 mM biocytin hydrazide (Thermo Fisher Scientific, for *ex vivo* recordings) or ~12 mM neurobiotin (Vector Laboratories, for *in vivo* recordings) was added to the internal solution to label recorded cells. The osmolarity of the final internal solution was adjusted 265–280 mOsm/kg by adding potassium aspartate. For *ex vivo* recordings, brains of ≧2 day old flies were excised from the head capsule in extracellular saline and the perineural sheath above the ePN somata was removed with fine forceps. For *in vivo* recordings, the dorsal side of ≧2 day old female flies was restrained with gel epoxy on a plastic film with a small window over the fly’s head. The dorsal side of the fly was immersed in extracellular saline and the dorsal cuticle between the compound eyes was removed. Both the antennae and maxillary palps were kept dry over the course of experiments. Fat tissue and tracheae over the ALs were removed and the muscle running along the esophagus was cut at the anterior side of the brain. The perineural sheath over the ePN somata was removed with fine forceps. The composition of the extracellular saline was as follows: 103 mM NaCl, 3 mM KCl, 5 mM N-[Tris(hydroxymethyl)methyl]-2-aminoethanesulfonic acid, 10 mM trehalose, 10 mM glucose, 26 mM NaHCO_3_, 1 mM NaH_2_PO_4_, 1.5 mM CaCl_2_, and 4 mM MgCl_2_. pH was equilibrated around 7.3 by bubbling with 95% O_2_/5% CO_2_. The osmolarity of the external solution was ~280 mOsm/kg without adjustment. The flow rate of perfusion was set at 2 mL/min by a RP-1 peristaltic pump (RAININ) and the saline temperature was maintained at 23–24°C by TC-324B, SH-27B and TS-70B inline heater and thermistor (Warner Instruments). The electrode was directed to the anterodorsal ePN soma under visual guidance using an Axio Examiner.A1 (Zeiss) microscope with a ×40 water-immersion objective. ePNs were identified by the location of their somata and their characteristically small spike size (~10 mV). Recordings were acquired with a MultiClamp 700B (Molecular Devices) amplifier and signals were low-pass filtered at 10 kHz and digitized at 20 kHz by a Digidata 1440B (Molecular Devices) digitizer. The resting membrane potential was kept ~ −58 mV by injecting a small hyperpolarizing current in current clamp mode. Voltages were not corrected for the liquid junction potential.

### Optogenetics

Flies were cultured on standard fly food covered with potato flakes rehydrated with 1.75 mM all trans-Retinal (ATR). ATR was prepared as 35 mM in ethanol as a stock and diluted for use 20-fold. For flashes of whole-field illumination of 590 nm light, the shutter on the Colibri LED light source was controlled using the Micro Toolbox (Zeiss). For localized stimulation of 590 nm light on the AL, ventrolateral protocerebrum (vlpr) and LH, illumination patterns generated in PolyScan2 or PolyLite software (Mightex) were flashed on the brains using the BioLED Light Source Control Module and Polygon400 Dynamic spatial illuminator (Mightex). Both the shutter of Colibri and BioLED Light Source Control Module were triggered with TTL input from a Master-8 (A.M.P.I) timer, which was synchronized with data acquisition by the Digidata1440B.

### Pharmacology

TTX, PTX, CdCl_2_ and histamine were prepared as 2 mM, 5 mM, 100 mM and 100 mM stocks in distilled water and diluted at 1:2000, 1:500, 1:1000 and 1:1000, respectively, for use. CGP54626 was prepared as a 50 mM stock in DMSO and diluted at 1:1000. Drug solutions were perfused for about 5 min (~10 mL) before their effects were tested. To confirm that genetically expressed His-Cl channels effectively suppress *MZ699-Gal4* expressing neurons, we expressed both His-Cl channels and CsChrimson in *MZ699-Gal4* neurons. Strong EPSPs evoked in ePNs were effectively reduced in the presence of 100 μM histamine (data not shown).

### Odor Stimulation

Benzaldehyde, 2-Octanone, Pentyl acetate, Ethyl acetate and Ethyl butyrate were diluted at 0.3% v/v with mineral oil in glass vials. A vial with only mineral oil and an empty vial were included as negative controls. Two hundred mL/min air flow filtered through activated charcoal was directed to the fly through a 3 mm i.d. tube whose outlet was set 8 mm away from the fly. One hundred mL/min air flow was pulsed by a PV820 pneumatic Picopump (World Precision Instruments), which was triggered by a Master-8 (A.M.P.I) timer, filtered through activated charcoal, and passed through an odor bottle. Tubes delivering the odorized air were merged into the constant flow 16 cm away from the outlet of the tube delivering the constant flow to the antennae. The 100 mL/min air flow was manually switched with a custom-made olfactometer and directed to one of seven vials. Odor pulse duration was 1 s and pulses were presented at 40 s intervals. For recordings in the antennal-less preparation, the odors ethyl acetate and ethyl butyrate, which typically induce strong lateral excitation in antennal ePNs, were mainly used. For recordings in preparations with intact antennae and palps, the five odors listed above were used to pre-test preparations before recordings began, and odors that elicited an increase in a spike rate were then used for the following recordings in the absence or presence of histamine.

### Immunohistochemistry

Brains used for recordings were fixed in 4% paraformaldehyde in Sorenson’s buffer (0.2 M, pH 7.2) for 30 min at room temperature. Brains were washed in Sorenson’s buffer, incubated in 10% goat serum in 0.3% Triton X-100 in Sorenson’s buffer (PBT) for 1 h at room temperature, and then incubated in 1:5 or 1:10 anti-Bruchpilot antibody (nc82, Developmental Studies Hybridoma Bank) and 1% goat serum in PBT for 2 days at 4°C. Brains were washed in PBT for 10 min at room temperature four times and then incubated in PBT containing 1:500 rabbit anti-GFP-Alexa488 (catalog #A-21311, ThermoFisher Scientific), 1:100 or 1:200 goat anti-mouse Alexa Fluor 568 (catalog #A-11031), 500 μg/L streptavidin Alexa Fluor 633 (catalog #S21375) and 1% goat serum for 2 days at 4°C. After washing in PBT for 10 min at room temperature four times, brains were mounted on a glass slide with 70% glycerol in Sorenson’s buffer.

### Data Analysis

Analyses were performed using custom programs written in MATLAB (MathWorks). To measure EPSP amplitude and effects of GABA, voltage traces were low-pass filtered at 13 Hz to remove spikes. The baseline membrane potential (1 s period prior to the light stimulus or GABA pulse) was then subtracted from the whole trace to compute deflections from the baseline. To compute EPSP latency from the light onset a second order parabolic function was fit to the EPSP, and EPSP onset was defined as the time this function intersected with the baseline (voltage recorded during a 1 ms period before the light stimulus). Peri-stimulus time histograms (PSTHs) were calculated to characterize odor-elicited spiking responses *in vivo*. Spikes trains for each trial were binned (50 ms bins overlapping by 25 ms), and then, for each trial, the average spike rate during a 2 s period prior to the odor stimuli was subtracted from each bin. Bins were then averaged across trials to yield an average PSTH. From this, the maximum spike rate was calculated from the bin with the highest count, and the total spike count was computed as the area under the curve of the PSTH. Multiple comparisons were corrected with Bonferroni’s method.

## Results

### vPNs Strongly and Reliably Excite ePNs

*MZ699-Gal4* is expressed in about 90% of vPNs, about 80% of which are GABAergic (Lai et al., 2008; Okada et al., 2009). It is not known which neurotransmitter the remaining 20% of MZ699-vPNs express. To characterize connections from vPNs to ePNs we used an optogenetic technique: we expressed CsChrimson driven by *MZ699-Gal4* in vPNs, and activated them with 200 ms of whole-field 590 nm light (~64 μW/mm^2^ corresponding to 5% intensity of our whole-field light stimulation) while performing whole-cell patch clamp recording from ePNs. This light stimulus induced huge depolarizations in a MZ699-vPN well above the spiking threshold (Figure 1C). Because the great majority of vPNs are GABAergic we predicted that activating them as a population would inhibit the ePNs. Surprisingly, vPN activation reliably excited ePNs (Figure 1C); when we activated vPNs this way, we never observed inhibition in an ePN. The magnitude of excitation was generally much stronger than would be expected from an electrical synapse (Wang et al., 2014) and varied by glomerulus (Figure 1D). The magnitudes of these EPSPs were light-dose dependent and saturated at around 5% of the maximum intensity of our whole-field light source. A 200 ms stimulus sufficed to induce maximal EPSPs (Figure 1E). Whole-field 200 ms 590 nm light did not excite ePNs in animals of the same genotype that were not supplemented with ATR (1.05 ± 0.29 mV of excitation, *n* = 5).

We sought to localize the source of excitatory input to the ePNs. *MZ699-Gal4* (and thus CsChrimson) is also expressed in a second large group of cells, the vlpr neurons, located in the vlpr (Figures 1A,B). These neurons may make direct contact with axon terminals of ePNs in the LH (Figure 1B), and could therefore be a source of the light-driven excitatory input to the ePNs. To test this possibility, we used a digital mirror device (DMD) to selectively illuminate the preparation, directing a narrow circle of 590 nm light centered on the AL, LH or vlpr (Figure 2A). The magnitude of excitation observed when we illuminated the AL was significantly much larger than when we illuminated the LH or vlpr (Figure 2B), indicating that the source of the EPSPs in the ePNs upon activation of *MZ699-Gal4* positive neurons is located in the AL. We more precisely localized the source of excitation to ePNs by directing narrower beams of light to the brain. In all six ePNs recorded, illuminating within the AL induced the largest EPSPs, while illuminating areas devoid of *MZ699-Gal4* expression induced significantly smaller EPSPs (Figures 2C,D, 21.67 ± 4.14% of the peak amplitude in the AL, *n* = 6). Close examination of the *MZ699-Gal4* expression pattern in the AL revealed two labeled fibers innervating the AL in addition to vPN neurites (Figures 2E1,E2). To test if these additional fibers are the source of excitation to the ePNs, we used an intersectional genetic approach (*MZ699-Gal4* and *Gad1-LexA::QFAD2*) so that activating expression of Gal80 resulted in suppressing expression of CsChrimson only in vPNs (Figures 2E3,E4). When we activated the two additional fibers with light flashes, we found that the magnitude of excitation in ePNs was significantly lower than that on activation of *MZ699-Gal4* positive neurons in corresponding glomeruli (Figures 2F,G). Together, these results demonstrate that vPNs excite the ePNs in the AL, probably through dendrodendritic connections.

### vPNs Are Monosynaptically Connected to ePNs through Chemical Synapses

vPNs have previously been shown to evoke small excitatory potentials in ePNs through bidirectional electrical synapses composed of shakB containing gap junctions (Wang et al., 2014). To further characterize vPN-ePN connections, we optogenetically activated CsChrimson-expressing vPNs with brief (100% wide field 590 nm light, 2 ms) flashes of light and measured the latency from the onset of the light flashes to the onset of EPSPs in ePNs. This method of measuring the latency from light onset to onset of EPSCs to establish monosynaptic connectivity from channel rhodopsin (ChR) expressing cells to recorded cells is well-established (Kohara et al., 2013; Root et al., 2014). All ePNs examined this way showed EPSPs of short latency (*n* = 5, 1.33 ms ± 0.13 ms), consistent with monosynaptic connectivity (Figures 3A,B). These short latency EPSPs could comprise both fast potentials through electrical synapses followed by slower chemical polysynaptic potentials from unknown neurons. To test this possibility, we blocked chemical synaptic release by bathing the preparation with Cd^2+^. By subtracting the residual Cd^2+^-insensitive voltage traces from the traces recorded before drug application we could reveal any purely chemical transmission component. We found the response latency of the remaining chemical component still had a short latency (1.27 ms ± 0.10 ms, *n* = 5, Figures 3A,B). These results rule out the possibility that electrical synapses alone underlie fast EPSPs from MZ699-vPNs to ePNs, and are consistent with ePNs receiving monosynaptic chemical excitation from the vPNs.

To further test whether these connections were monosynaptic, we blocked polysynaptic transmission by bathing the preparation with TTX. Blockade of voltage-gated Na^+^ channels has been used previously to establish monosynaptic connections from ChR expressing cells to recorded cells (Franks et al., 2011; Sugimura et al., 2016). We found that EPSPs in ePNs elicited by optogenetic activation of vPNs persisted even in the presence of TTX, though the EPSPs were slightly smaller in amplitude (78.54 ± 5.45% of pre-drug, *n* = 5, *p* = 0.027, Figures 3C–E). Because TTX blocks the generation of action potentials by voltage-gated Na^+^ channels, these optogenetically induced EPSPs in ePNs were caused by CsChrimson-triggered synaptic release from vPN presynaptic sites. When TTX and Cd^2+^ were both added to the bath, residual EPSPs were greatly and significantly reduced (18.48 ± 4.13% of pre-drug, *n* = 5, *p* = 0.0030, Figures 3C–E), indicating that they were induced largely through chemical transmission. It is possible that local circuits (Berck et al., 2016) could mediate indirect connections between vPNs and ePNs; for example, optogenetically activated vPNs could excite Chrimson negative neurons which then excite ePNs in a spike-independent manner. However, our pharmacological manipulations combined with our measurements of very brief EPSP latencies make this scenario unlikely, and rather suggest that vPNs mainly excite ePNs through monosynaptic chemical synapses.

### GABA May Not Contribute to vPN-ePN Excitatory Transmission

What transmission mechanism allows predominantly GABAergic vPNs to excite ePNs? GABA is a major inhibitory neurotransmitter in the *Drosophila* central nervous system; it causes inhibition by activating GABA_A_ and GABA_B_ receptors, leading to the influx of Cl^−^ ions and efflux of K^+^ ions, respectively (Sodickson and Bean, 1996; Hosie et al., 1997). GABA is also known to depolarize postsynaptic neurons in some circuits (notably in the developing mammalian brain) where elevated intracellular concentrations of Cl^−^ shift the Cl^−^ reversal potential such that activating GABA_A_ receptors leads to a depolarizing efflux of Cl^−^ ions. However, we found that the amplitude of EPSPs triggered in ePNs by photoactivation of CsChrimson expressing MZ699-vPNs was not affected by bath application of 10 μM PTX (*n* = 5, *p* = 0.059, Figures 4A,B), a treatment that effectively blocks GABA_A_ receptors in *Drosophila* (Olsen and Wilson, 2008). This suggests vPN-triggered EPSPs in ePNs are not mediated by GABA_A_ receptors. Further, our intracellular patch solution contained low concentrations of Cl^−^ ion making it unlikely that the Cl^−^ reversal potential of recorded neurons favored their depolarization by GABA (Raimondo et al., 2017). GABA has been shown to activate other types of receptors, potentially leading to excitation. For example, it has been reported that GRD (GABA and glycine-like receptor of *Drosophila*) and ligand-gated chloride channel homolog 3 (LCCH3) form GABA-gated heteromultimeric cation channels in the *Xenopus laevis* oocyte heterologous expression system. However, GABA-elicited inward flow of cations in oocytes expressing GRD and LCCH3 could also be blocked by application of 10 μM PTX (Gisselmann et al., 2004), excluding the possibility that GRD and LCCH3 underlie the excitatory input from MZ699-vPNs to ePNs.

To test if GABA synthesis in the MZ699-vPNs is necessary for the depolarization of ePNs, we used RNAi to knock down the *Gad1* gene in neurons expressing *MZ699-Gal4*. We then compared the amplitudes of EPSPs triggered in ePNs by photoactivation of CsChrimson expressing MZ699-vPNs between control and RNAi animals. Although we found that GABA immunostaining was significantly reduced in RNAi MZ699-vPNs (Figure 4C, *n* = 8 hemispheres for control, *n* = 8 hemispheres for RNAi, *p* < 0.001, *t*-test), EPSP amplitude on photoactivation of MZ699-vPNs was not reduced in the nine glomeruli we tested (Figure 4D, *n* = 25 for control, *n* = 21 for RNAi, *p* = 0.59, two-way ANOVA).

We further tested whether pressure ejecting GABA over the desheathed AL causes depolarizing responses in ePNs. In a normal saline bath, GABA pulses reliably hyperpolarized the ePNs, indicating that the GABA pulses were effectively delivered to the recorded cells (Figure 4E, top). Since hyperpolarizing potentials mediated by GABA_A_ and GABA_B_ receptors could mask a possible excitatory response, we then pulsed GABA in the presence of 10 μM PTX and 50 μM CGP54626, a GABA_B_ receptor antagonist effective in *Drosophila* (Wilson and Laurent, 2005). In the presence of the antagonists, we never observed excitatory responses to GABA (Figure 4E bottom, Figure 4F, *n* = 5, −7.57 ± 1.56 mV before antagonists, 0.19 ± 0.10 mV after antagonists). All the above results suggest GABA does not mediate the excitatory transmission at vPN-ePN synapses.

### vPN-ePN Excitatory Connections May Not Underlie Wide-Spread Lateral Excitation in the AL

What is the functional significance of the direct vPN-ePN chemical synapses? We hypothesized that vPNs could contribute to lateral excitatory interactions among glomeruli because a subset of vPNs has multiglomerular branches in the AL and because MZ699-vPNs, as a group, strongly excite ePNs. In the *Drosophila* AL, odor-elicited lateral excitatory interactions among glomeruli persist in preparations in which both antennal nerves were acutely severed, and thus lack direct afferent inputs from the antennae to the antennal ePNs (Figure 5A). These lateral excitatory interactions have been reported to spread through shakB-dependent electrical synapses between ePNs and two or three *krasavietz-Gal4* expressing excitatory LNs (eLNs; Yaksi and Wilson, 2010). To compare the possible roles of shakB-dependent electrical synapses and vPN-ePN excitatory connections, we tested odor-evoked lateral excitatory inputs to antennal ePNs in several glomeruli in *shakB*^2^ mutants, which lack the neural isoform of shakB innexin molecules (Zhang et al., 1999). Consistent with a previous report (Yaksi and Wilson, 2010), lateral inputs to antennal ePNs in two glomeruli, DA1 and VA5, were greatly reduced and appeared to be dominated by inhibition. This trend was observed in all other glomeruli in the same lateral ePN lineage as DA1 and VA5 (for ethyl acetate, peak depolarization was 5.71 ± 1.89 mV in control flies and 0.76 ± 0.67 mV in *shakB^2^* mutants. For ethyl butyrate, peak depolarization was 6.23 ± 2.60 mV in control flies and 0.48 ± 0.46 mV in *shakB^2^* mutants; *n* = 13 for control and *n* = 6 for *shakB^2^* mutant including ePNs in DA1 and VA5, *p* < 0.001, Wilcoxon rank sum test). However, in two other glomeruli, VC3 and VM5v, in the same *shakB*^2^ mutants we observed large amplitude odor-elicited depolarizations (Figure 5B). These results indicate that shakB-dependent electrical synapses between ePNs and eLNs do not provide the sole mechanism underlying lateral excitatory interactions in the *Drosophila* AL.

To test whether vPNs contribute to these lateral excitatory responses, we suppressed their activity by expressing shunting His-Cl channels in *MZ699-Gal4* neurons. Prior work showed that application of histamine by itself does not affect the odor responses, resting potentials, or input resistance of ePNs in *Drosophila* (Liu and Wilson, 2013b). We found that suppressing vPNs by providing histamine in the bath did not reduce the amplitudes of odor-evoked EPSPs in ePNs (Figures 6A–C, 6 ePNs in 4 glomeruli, 7 odor-ePN pairs, *p* = 0.23 for the peak amplitude, *p* = 0.14 for the area under the curve, paired *t-test*). We also tested whether odor-elicited responses of ePNs in animals with intact antennae and maxillary palps were reduced by suppressing MZ699-vPNs. Though decreases both in peak response spike rate and total response spike count were seen in a few odor-ePN pairs, the overall trend across many glomeruli was that suppression of MZ699-vPNs did not change the odor response of ePNs (Figures 6D–G, 13 ePNs, 32 odor-ePN pairs, *p* = 0.53 for the peak response rate, *p* = 0.13 for the total response spike count). An increase in odor responses in some glomeruli is consistent with our observation that inhibitory local neurons (iLNs) also receive excitatory inputs from MZ699-vPNs (data not shown); decreased activation of iLNs on suppression of MZ699-vPNs may have disinhibited the odor responses of ePNs. These results suggest the MZ699-vPNs are unlikely to underlie wide-spread lateral excitatory interactions in the *Drosophila* AL.

## Discussion

In the insect olfactory system, odor information detected on the olfactory appendages is processed in the AL and is then sent to the MB and LH through multiple antennal lobe tracts (ALTs, Schachtner et al., 2005). In flies and moths, the mALT targets the MB calyx and the LH, and the mlALT targets only the LH, bypassing the calyx (Homberg et al., 1988; Stocker et al., 1990; Tanaka et al., 2012). In honeybees, on the other hand, the mALT and lALT target the MB calyx and LH, and the mlALT directly targets the LH (Kirschner et al., 2006). In all these species, despite the slight differences in the projection patterns of ALTs, a substantial proportion of mlALT neurons are GABAergic (Hoskins et al., 1986; Schäfer and Bicker, 1986; Okada et al., 2009). In *Drosophila*, *in situ* hybridization analysis and genetic labeling strategies have revealed that about 80% of MZ699 expressing vPNs are GABAergic, and appear to have relatively few presynaptic sites in the AL (Okada et al., 2009; Liang et al., 2013). These observations have helped draw attention to the roles inhibition from vPNs may play upon their postsynaptic partners in the LH (Liang et al., 2013; Parnas et al., 2013; Wang et al., 2014). However, very little is known about the properties and functions of synapses vPNs make with their dendritic branches in the AL although it has been reported that vPNs form weak electrical synapses with ePNs through gap junctions (Wang et al., 2014).

We used optogenetic and pharmacological manipulations to investigate synapses between vPNs and ePNs in the AL. We found that in the AL, vPNs labeled by the Gal4 driver *MZ699-Gal4* form monosynaptic, excitatory chemical synapses onto ePNs. When driven optogenetically, vPNs depolarize ePNs in several glomeruli, with the amplitudes of the EPSPs relatively large and varying with the glomerulus (Figure 1D). *MZ699-Gal4* is expressed not only in vPNs but also in neurons in other locations in the brain, including the vlpr neurons and two fibers innervating the AL, both of which could potentially make direct contacts with ePNs (Figures 1A,B, 2E1,E2). However, by selectively illuminating different brain areas and restricting CsChrimson expression through a genetic intersectional approach, we determined that the MZ699-vPNs are the dominant source of excitation to the ePNs (Figure 2). We further determined that connections from MZ699-vPNs to ePNs were monosynaptic by measuring the time between the onset of photoactivating light flashes and the onset of purely chemical component of EPSPs in ePNs (<1.5 ms), and by comparing the light-evoked EPSP amplitude before and after blocking action potentials with bath-applied TTX (Figure 3).

Although GABA most often mediates inhibition, it is known to play excitatory roles in some contexts, such as in immature mammalian brains, in worms, and in the oocyte heterologous expression system with *Drosophila* GRD/LCCH3 (Beg and Jorgensen, 2003; Gisselmann et al., 2004; Raimondo et al., 2017). However, multiple lines of evidence suggest GABAergic transmission cannot explain synaptic excitation in the MZ699-vPNs to ePNs synapses (Figure 4): (1) the amplitude of EPSPs in ePNs on activation of MZ699-vPNs was not reduced by application of PTX, which effectively blocks both GABA_A_ receptors and GRD-LCCH3 cation channels in *Drosophila*; (2) RNAi knock down of the GABA-generating enzyme Gad1 in *MZ699-Gal4* positive cells did not reduce EPSP magnitude in ePNs; and (3) exogenous application of GABA pulses to the AL in the presence of GABA_A_ and GABA_B_ antagonist never induced depolarization in ePNs.

About 20% of MZ699-vPNs are unlikely to be GABAergic because they were not labeled by a DNA *in situ* hybridization probe against *Gad1* mRNA (Okada et al., 2009). If these vPNs express an excitatory neurotransmitter whose synaptic release activates corresponding receptors in the ePNs, the excitatory vPN-ePN synaptic connections we observed could potentially be mediated by these vPNs. Glutamate and acetylcholine are the most common fast neurotransmitters in the insect peripheral and central nervous system, respectively (Osborne, 1996). In *Drosophila*, however, iontophoresis of glutamate into the AL has been shown previously to inhibit the ePNs through glutamate-gated Cl^−^ channels (Liu and Wilson, 2013a). We further found that bath application of mecamylamine (a nicotinic acetylcholine receptor antagonist) did not reduce depolarization of ePNs upon optogenetic activation of MZ699-vPNs (data not shown, although other cholinergic receptors insensitive to mecamylamine could mediate excitatory cholinergic transmission from vPNs to ePNs). It is also possible that excitatory transmitters other than glutamate or acetylcholine that are not well characterized in *Drosophila* may contribute to the depolarization of ePNs. Identification of expression lines that specifically label GABAergic or non-GABAergic subset of vPNs will allow further insights into synaptic mechanisms underlying the excitatory connection from vPNs to ePNs.

A possibility we cannot rule out is that GABAergic MZ699-vPNs could co-express an excitatory neurotransmitter and release it at specific compartments within cells; for example, GABA could be released at the axonal terminals in the LH and an excitatory neurotransmitter released at the dendritic presynaptic terminals in the AL. Indeed, several examples of mammalian neurons that can release multiple fast excitatory or inhibitory neurotransmitters have been reported, such as spatially segregated release of GABA and ACh in the retina (Lee et al., 2010; Vaaga et al., 2014; Granger et al., 2016).

Electrical synapses between *krasavietz-Gal4* eLNs and ePNs have been proposed to mediate lateral excitation between glomeruli in the *Drosophila* AL (Yaksi and Wilson, 2010), but several lines of evidence align against this mechanism. Because the number of eLNs is small (two or three), and the coupling reported between each eLN and ePN is weak (1–2 mV EPSPs), it is not clear how activity mediated by electrical synapses is sufficient to explain the strong odor-evoked lateral excitation observed in some glomeruli (~8 mV on average, up to 20 mV, Olsen et al., 2007, and Figure 5). In addition, recordings from eLNs in antenna-less preparations revealed odor responses that began with a period of inhibition, while responses of antennal ePNs (now lacking direct afferent input from antennal ORNs) began with a period of excitation (Kazama et al., 2011); if lateral excitation is mediated by the eLN-ePN electrical synapses, the sign of responses in ePNs and eLNs should match: the responses of eLNs also should begin with a period of excitation in the antenna-less preparation. Further, we found that mutants lacking shakB still showed strong odor-elicited lateral excitation in some glomeruli (Figure 5). Because some MZ699-vPNs form multiglomerular arborizations (Lai et al., 2008) and because we observed strong excitatory chemical connections from the MZ699-vPNs to the ePNs, MZ699-vPNs seemed well-positioned to mediate wide-spread lateral excitation. Indeed, a previous study proposed that vPNs could contribute to lateral excitation given that MZ699-vPNs make electrical synapses onto ePNs (Wang et al., 2014). However, we observed reduction of odor-elicited responses in neither antenna-less nor intact preparations when MZ699-vPN activity was suppressed by activating His-Cl channels (Figure 6). Thus, vPN-ePN excitatory connections are unlikely to underlie widespread lateral excitation.

vPN-ePN connections more likely mediate targeted, localized excitation in the AL. One possibility is that the strong synaptic connections revealed by our optogenetic experiments are mediated mostly, if not entirely, by uniglomerular vPNs; synapses from multiglomerular vPNs onto ePNs may be relatively weak; such a connectivity scheme could allow for amplifying the AL’s responses to particular odorants. We cannot at present differentiate these populations since the MZ699-vPN population we photoactivated includes both uniglomerular and multiglomerular vPNs. Identification of new Gal4 lines that label morphological subpopulations (uniglomerular or multiglomerular and different glomerular projections) of the vPNs will allow further insights into the roles of this population in olfactory information processing.

## Author Contributions

KS and MS: conceptualization; funding acquisition; KS: methodology; investigation; writing—original draft; MS: writing—review and editing; supervision.

## Funding

This work was funded by an intramural grant from the National Institutes of Health–National Institute of Child Health and Human Development (MS). KS was supported by the Japan Society for the Promotion of Science Research Fellowship for Japanese Biomedical and Behavioral Researchers at National Institutes of Health.

## Conflict of Interest Statement

The authors declare that the research was conducted in the absence of any commercial or financial relationships that could be construed as a potential conflict of interest.
