# Corrigendum: A Population of Projection Neurons that Inhibits the Lateral Horn but Excites the Antennal Lobe through Chemical Synapses in *Drosophila*

**DOI:** 10.3389/fncir.2018.00049

**Published:** 2018-06-20

**Authors:** Kazumichi Shimizu, Mark Stopfer

**Affiliations:** National Institute of Child Health and Human Development, National Institutes of Health, Bethesda, MD, United States

**Keywords:** olfaction, *Drosophil*a, antennal lobe, electrophysiology, electrical synapses, chemical synapses, GABA, lateral excitation

We have identified two errors in text symbols:

(1) In the legend of **Figure 1 (C)**, the unit of light intensity used for optogenetic stimulation is incorrect: All instances of “mW/mm^2^” in this legend should be changed to “μW/mm^2^”. Correct text will be as follows:

Optogenetic activation of *MZ699-Gal4* neurons with ~64 μW/mm^2^ 200 ms whole-field 590 nm light elicited large depolarizations well above the spiking threshold in a MZ699 vPN (left); the average (black) of 10 trials (gray) from an example MZ699-vPN. The spikes the recorded neuron produced were small and difficult to see in the raw traces. A raw voltage trace of the same neuron upon stimulation with ~1.3 μW/mm^2^ light for 200 ms is shown in the inset. A stronger light stimulus (~64 μW/mm^2^ 200 ms whole-field 590 nm light) delivered to the brain elicited large and reliable excitatory postsynaptic potentials (EPSPs) in the recorded ePNs (right); the average (black) of 10 trials (gray) from an example ePN in VM5v glomerulus is shown.

(2) In the subsection “Whole-Cell Patch Clamp Recordings” of section “MATERIALS AND METHODS”, in two places “≦2 day” should be changed to “≧2 day”. Correct text will be as follows:

For *ex vivo* recordings, brains of ≧2 day old flies were excised from the head capsule in extracellular saline and the perineural sheath above the ePN somata was removed with fine forceps. For *in vivo* recordings, the dorsal side of ≧2 day old female flies was restrained with gel epoxy on a plastic film with a small window over the fly's head.

The authors apologize for the mistakes. These errors do not change the scientific conclusions of the article in any way.

The original article has been updated.

## Conflict of interest statement

The authors declare that the research was conducted in the absence of any commercial or financial relationships that could be construed as a potential conflict of interest.

